# Burnout syndrome and minor mental disorders in public employees

**DOI:** 10.47626/1679-4435-2023-802

**Published:** 2023-04-18

**Authors:** Liliana Andolpho Magalhães Guimarães, Alessandra Laudelino Neto, João Massuda Júnior, Mariana Mateus Sartoratto, Millene Soares Cardoso

**Affiliations:** 1 Laboratório de Saúde Mental e Qualidade de Vida no Trabalho Universidade Católica Dom Bosco, Campo Grande, MS, Brazil; 2 Instituto Federal de Mato Grosso do Sul, Campo Grande Campus, Campo Grande, MS, Brazil

**Keywords:** mental health, professional burnout, occupational health, saúde mental, esgotamento profissional, saúde do trabalhador

## Abstract

**Introduction:**

The context of transformations the society has been going through, especially considering economic, political, and technological issues, has added strains to modern work relations.

**Objectives:**

This study aimed to assess the existence and levels of burnout and the prevalence of minor mental disorders in a sample of public administrative employees of the Social Security Agency of Mato Grosso do Sul, in Brazil.

**Methods:**

This is a cross-sectional study that used the following research instruments: the Maslach Burnout Inventory, the Self-Reporting Questionnaire, and a sociodemographic and occupational questionnaire developed specifically for this study.

**Results:**

The results showed a prevalence of 23.7% (n = 9) of suspected cases of minor mental disorders and extremely increased levels (91.4%) of one of the dimensions of burnout: reduced professional efficacy. Employees with suspected minor mental disorders presented higher levels of emotional exhaustion and lower levels of personal accomplishment.

**Conclusions:**

In addition to the reported evidence, we expect our findings to contribute to the development of strategies of preventive intervention and health promotion in this occupational sector.

## Abstract (pt)

**Introdução:**

O contexto de transformações pelas quais a sociedade vem passando, sobretudo relacionadas a questões econômicas, políticas e tecnológicas, tem tensionado, contemporaneamente, as relações de trabalho.

**Objetivos:**

O presente estudo objetivou avaliar a existência e os níveis da síndrome de *burnout* e a prevalência de transtornos mentais menores em uma amostra de servidores públicos administrativos da Agência de Previdência Social de Mato Grosso do Sul, no Brasil.

**Métodos:**

Foi realizado um estudo transversal, e os seguintes instrumentos de pesquisa foram utilizados: o Inventário de *Burnout* de Maslach, o Self-Report Questionnaire e um questionário sociodemográfico e ocupacional, desenvolvido especialmente para este estudo.

**Resultados:**

Os resultados evidenciaram haver prevalência de 23,7% (n = 9) como casos suspeitos de apresentarem transtornos mentais menores e níveis extremamente acentuados (91,4%) em uma dimensão do *burnout*: baixa realização profissional. Servidores considerados suspeitos de apresentarem transtornos mentais menores apontaram maiores níveis de exaustão emocional e menores níveis de realização pessoal.

**Conclusões:**

Para além das evidências relatadas, espera-se que os achados cooperem para o desenvolvimento de estratégias de intervenção preventivas e promotoras de saúde nesse segmento ocupacional.

## INTRODUCTION

The transformations the society has been through in recent years and technological advances have led public and private organizations to search for new management options, with the aim of improving their team’s performance and optimizing their units’ results. In some work environments, the increased pressure to achieve ever more challenging goals has not only made workers increase their work pace but has also overstepped previously determined limits of the workday, exposing them to practices and behaviors that have harmful effects to their physical and mental health.^1^

In the occupational mental health domain, continuous exposure to an excessive workload, the lack of training for performing the requested tasks, the absence of social support, a low level of control over the performed activity, and work-rest periods incompatible with biological limits, among other reasons, may trigger a dysfunctional reaction in workers associated with a feeling of excessive demands, with the impression of depletion of physical and/or emotional resources.^2-5^ In Brazil, public institutions usually lack adequate management instruments and strategies, with frequently centralized decision-making and paternalism, outside political interference, and discontinuity in management and projects, which tends to generate pressure and stress.^6^

Studies performed by different authors^5,7-9^ suggest that continued exposure to occupational stress factors, which are usually silent, under the influence of work characteristics and institutional factors for its development, may lead to burnout syndrome. This syndrome, in a psychosociological perspective, is based on the presence of three dimensions: (i) emotional exhaustion (a response to chronic stress, involving feelings of depletion of emotional resources); (ii) depersonalization (refers to the worker’s emotional hardening, avoiding involvement with those he or she interacts with); and (iii) reduced professional efficacy (a self-reported component related to reduced quality and competence considering the performed work).^8^

In 2019, the World Health Organization (WHO) classified burnout as an occupational phenomenon of social and economic importance, as it relates to increased absenteeism and a higher demand for health care services.^5^ Burnout presents itself as a psychosocial phenomenon permeated by numerous economic, political, and technological changes, which has ultimately added strains to work relations.^10^ In Brazil, although the Ministries of Health and Social Security recognize burnout as a work-related occupational disease, its notification is still rare due to its poor understanding within the social security sector.^11^

Throughout the last decades, studies have progressively shown relationships between psychosocial working conditions and the mental health of workers,^12^ with impacts on their reality, their families, employers, health systems, and society in general.^13^ It is important to look closely at the health of workers and the mental suffering and strain that may affect their work.

The WHO clarifies that approximately 30% of active workers are affected by minor mental disorders (MMD).^14^ Some studies show that workers who go through MMD tend to delay seeking specialized care or, many times, do not request it at all.^15,16^

On the other hand, MMD correspond to a set of non-psychotic symptoms of insufficient intensity to characterize a specific mental disorder (such as insomnia, fatigue, irritability, forgetfulness, difficulty concentrating) and somatic complaints.^17^ These symptoms and complaints do not implicate in the nosological diagnoses proposed by international classifications, even though these disorders are recognized as one of the main public health problems worldwide. Mental suffering, decreased quality of life, sickness absenteeism, premature retirement, premature death, and other depressive disorders are some of the harmful effects frequently associated with the presence of MMD in workers.^18^ A study with state employees^19^ revealed the wide influence of mental disorders as the cause of leaves and the pressing need for epidemiological studies in this population.

Studies that measure the correlation between professional burnout syndrome and MMD indicate high social, economic, and individual costs as results.^20^ Therefore, we identified the need and the academic and social relevance of investigating the existence and levels of burnout and the prevalence of MMD, as well as their correlation in state administrative employees of the Social Security Agency of Mato Grosso do Sul (AGEPREV), in Brazil, also identifying sociodemographic and occupational variables.

## METHODS

This is a descriptive, cross-sectional study, with a quantitative approach and convenience sampling of 38 public administrative employees (73% of the staff) of AGEPREV, located at the municipality of Campo Grande, in the state of Mato Grosso do Sul.

The participants were informed of the study objectives and consented to participate in the study by signing an informed consent form (ICF) before answering the instruments. They were also informed that their information would be kept confidential and consent could be withdrawn at any moment just by requesting any of the professionals involved in data collection to be excluded from the study.

This study was previously approved by the Research Ethics Committee through Opinion No. 1718042, and all ethical rules were observed in its conduction.

For collecting data relative to the presence and levels of burnout and MMD in the studied sample, we used a sociodemographic and occupational questionnaire developed specifically for this study, as well as the translated and adapted version of the Maslach Burnout Inventory (MBI)^20^ and the translated version of the Self-Reporting Questionnaire (SRQ-20) validated to Brazil.^21^

The MBI was chosen for assessing burnout because this is the most commonly used scale nationally and internationally for detecting this syndrome, allowing the epidemiological assessment of burnout. SRQ-20 has been widely used in Brazil for assessing the level of suspicion of mental disorders in workers, leading to satisfactory results in many studies.^22^

The collected data were analyzed using SPSS software, version 25. The sociodemographic and occupational characteristics of the studied individuals were described through descriptive statistics and by analyzing frequency distributions. Additionally, we compared the means obtained in different groups through inferential statistics. We chose the independent samples t-test for evaluating the relationship between some of the studied variables, and a p-value of 0.05 or less was used as criterium for the analyses.

## RESULTS

The study participants were, for the most part, female (68.4%), married or in a common-law marriage (60.5%), had a mean age of 43.05 years (standard deviation [SD] 4.80), and had kids (68.4%); 86.9% of the respondents reported they had a higher education degree, 55.3% declared an individual monthly income of more than R$ 2,000.00 and less than R$ 5,000.00, and 34.2% indicated a household monthly income between R$ 5,000.00 and R$ 8,000.00; the study did not assess whether this income (individual or household) included earnings of on-call work or other work activities. Most participants (81.6%) also stated they slept between 5 and 8 hours a day, and 53.6% said they were physically active.

As to their occupational characteristics, 10.5% of the participants revealed they did on-call work, 7.9% informed they worked over hours, and 10.5% reported they had another job; this study did not assess the activity performed as a source of additional income.

Regarding the occupations of the study participants, 28.9% of respondents held management/director jobs, 18.4% performed administrative assistance activities, 15.8% were analysts, developers, programmers, or information technology technicians, 10.5% had advisory roles, and 26.3% performed various tasks. When it came to their work environment, 63.2% evaluated their relationships at work as good, 71.1% believed that work did not interfere with their family life, and 81.6% declared their family did not interfere with their work.

The SRQ-20 results indicated 23.7% of the respondents (n = 9) as suspected cases of presenting MMD. The frequency distribution of the participants’ answers to the instrument is described in Table 1.

**Table 1:** Frequency distribution of symptoms of minor mental disorders in the studied sample, according to the Self-Reporting Questionnaire instrument

Symptoms	SRQ-20 items	n	%
Depressive/anxious mood	Feels nervous, tense, or worried	22	57.9
	Is easily frightened	8	21.1
	Feels unhappy lately	10	26.3
	Cries more than usual	6	15.8
Somatic symptoms	Frequent headaches	12	31.6
	Uncomfortable feeling in the stomach	12	31.6
	Hand shaking	4	10.5
	Poor digestion	10	26.3
	Poor sleep	8	21.2
	Lack of appetite	2	5.3
Reduced vital energy	Difficulty making decisions	10	26.3
	Is easily tired	15	39.5
	Has trouble thinking clearly	5	13.2
	Has difficulties enjoying daily activities	10	26.3
	Feels tired all the time	8	21.1
	Work brings suffering	2	5.3
Depressive thoughts	Has lost interest in things	6	15.8
	Finds it hard to feel useful/important	-	-
	Feels useless/worthless	2	5.3
	Thinks about taking own life	-	-

n = 38 public administrative employees of the Social Security Agency of Mato Grosso do Sul (AGEPREV), Brazil; SRQ-20 = Self-Reporting Questionnaire.

Out of the symptoms measured by SRQ-20, we noticed a predominance (57.9%) of a depressive/anxious mood, especially in matters linked to nervousness, tension, and constant worrying.

The analysis of data collected through the MBI indicated that 97.1% of the participants presented low levels of emotional exhaustion, 100% reported low levels of depersonalization, and 91.4% informed they noticed a reduced professional efficacy. It is important to note that three participants of this study did not answer this instrument, which is why the analyses were done considering 35 respondents. The internal consistency assessment of the three instrument subscales indicated adequate levels for the subscale assessing emotional exhaustion levels (α = 0.87) and personal accomplishment (α = 0.74). The subscale dedicated to assessing depersonalization presented a low level of internal consistency (α = 0.30) (Table 2).

**Table 2:** Mean, standard deviation, and reliability of Maslach Burnout Inventory dimensions

Dimensions	n	Scale	Mean	SD	Alpha
Emotional exhaustion	35	1-5	2.26	0.77	0.87
Depersonalization	35	1-5	1.72	0.51	0.30
Personal accomplishment	35	1-5	3.39	0.56	0.74

n = 35 public administrative employees of the Social Security Agency of Mato Grosso do Sul (AGEPREV), Brazil; SD = standard deviation.

We carried out tests for comparing the means of independent samples for the dimensions of burnout according to the score obtained in the SRQ-20. The results indicated that individuals with suspected MMD evidenced higher levels of emotional exhaustion and lower levels of personal accomplishment when compared to workers without suspected MMD, and this difference was statistically significant (p < 0.001 and p = 0.005, respectively) (Table 3). The participants who reported a perception that their work interfered with their family life reported higher levels of emotional exhaustion and depersonalization when compared to respondents who did not notice this influence (Table 3).

**Table 3:** t-test for comparing the means of independent samples for the dimensions of burnout according to the score obtained in the screening of minor mental disorders by Self-Reporting Questionnaire.

MBI	SRQ-20		T^*^	dF†	Sig‡
	Not suspected Mean (SD)	Suspected Mean (SD)			
Emotional exhaustion	1.97 (±0.57)	3.11 (±0.66)	-4.924	33	0.000
Depersonalization	1.65 (±0.47)	1.93 (±0.59)	-1.435	33	0.161
Personal accomplishment	3.54 (±0.46)	2.95 (±0.62)	2.975	33	0.005

* Student’s t statistics.

† Degrees of freedom.

‡ Statistical significance of the test.

MBI = Maslach Burnout Inventory; n = 35 public administrative employees of the Social Security Agency of Mato Grosso do Sul (AGEPREV), Brazil; SD = standard deviation; SRQ-20 = Self-Reporting Questionnaire.

When comparing the mean scores obtained in the SRQ-20 for individuals who reported a possible interference of work in family life, we identified a statistically significant difference (p = 0.010) between scores of both groups; the one for the positive response (interferes) was 6.72 (±4.79) and the negative (does not interfere) was 2.88 (±3.59), with a t of 2.704 and a degree of freedom (dF) of 36. Respondents who reported an interference of work in family life presented higher mean SRQ-20 scores when compared to those in the other group; their scores were quite close to the cutoff score for identifying suspected cases of MMD (≤7).

## DISCUSSION

The presence of burnout among administrative employees of AGEPREV in the municipality of Campo Grande is evidenced by extremely high indices (91.4%) of reduced professional efficacy. This finding corroborates the results of other studies with employees of the public sector.^6^ This result, which reveals the self-assessment aspect of burnout, tends to highlight its identification in the context of work and institutional factors for its development.^5^ Studies indicated by the Jorge Duprat Figueiredo Foundation for Occupational Safety and Medicine (Fundacentro) stated that working conditions tend to affect the engagement of public employees more intensely than financial incentives.^23^

The prevalence of 23.7% for suspected MMD, in this study, was higher than that found in other studies with public employees^24,25^ - 20.3 and 16%, respectively -, but it was lower than that found in teachers and health professionals - 36.9 and 27.7%, respectively.^26^ Dedicated to detect and measure the level of suspicion (presence/absence) of mental disorders, the SRQ 20 screening instrument refers to and attributes importance to the care related to primary levels of health care.^27^

As to details of SRQ-20, the group of symptoms that identifies a depressive-anxious mood (Table 1) highlights that 57.9% of the public employees reported feeling nervous, tense, or worried. Similar data were found in the literature, emphasising this complaint as the most relevant in the group of symptoms in the SRQ-20.^27,28^

The associations established between workers with suspected MMD and burnout presented higher levels of emotional exhaustion indicators (3.11 [±0.66]; p < 0.001). It is possible to consider an association of the occurrence of MMD, characterized by fatigue, insomnia, irritability, difficulty concentrating, and somatic complaints, with the presentation of burnout-indicating components, such as feelings of emotional strain and lack of energy, revealing a trend of an inverse association with work performance.

As to the results of the association between employees who noticed an interference of work in family life and those who did not realize it, we found a statistically significant difference between scores obtained in burnout items related to emotional exhaustion (p = 0.006) and depersonalization (p = 0.032). In this sense, some authors^29^ clarify that studies relating the interference of work in family life (and vice-versa) and occupational health show that the dimensions of burnout are present in unbalanced relationships between the two areas of an individual’s life, not only as a consequence but as an indicator of displacement and exhaustion.

Discussions on the association between burnout and psychiatric morbidities have been held for decades. Both researched phenomena, in this study, can be seen as important in intensifying the workers’ mental strain. The results of this association point to the main factors that lead many employees to take leaves of absence.

Work-related mental health issues, with cases of stress, depression and anxiety symptoms, and burnout, is among the main factors that lead to leaves of absence among public employees; in the last 7 years, around 15 thousand federal employees took leaves of absence due to mental disorders, affecting public service as a whole.^23^

## CONCLUSIONS

This study evidenced high levels (91.4%) of the reduced professional efficacy dimension of burnout. The prevalence of suspected MMD was 23.7%, with a higher frequency of nervousness, tension, and constant worrying (57.9%).

We also observed that, the higher the frequency of MMD, the higher were the effects on the emotional exhaustion and professional efficacy dimensions of burnout. The emotional exhaustion and depersonalization dimensions of burnout presented a positive association with the workers’ perception that work interferes with their family life.

As limitations related with this investigation, we note the sampling type, which precluded generalizations. We suggest the conduction of studies with other units of the institution that encompass this occupational category. Nevertheless, the understanding brought by the study on some phenomena that act in the genesis of suffering and mental health-illness can cooperate to the development of strategies of preventive intervention and health promotion in this occupational sector.
